# Optimization of Phenylethylammonium Bromide Post-Treatment for 2D/3D Heterostructure Formation in High-Performance Perovskite Solar Cells

**DOI:** 10.3390/nano16211445

**Published:** 2026-11-08

**Authors:** Hui Liu, Guoquan Wu, Junqing Mo, Ruiheng Zhang

**Affiliations:** School of Physics and Opto-Electronic Engineering, Guangdong Provincial Key Laboratory of Sensing Physics and System Integration Applications, Guangdong University of Technology, Guangzhou 510006, China

**Keywords:** 2D/3D heterostructure, PEABr post-treatment, interface engineering, inverted perovskite solar cells

## Abstract

Inverted perovskite solar cells (IPSCs) have gained a lot of attention and a significant amount of research interest in recent years due to their outstanding optoelectronic properties and rapid enhancement of efficiency. However, the IPSCs are still significantly limited by the influence of abundant defects and detrimental nonradiative recombination, especially at the top interface of the perovskite film. Herein, a surface 2D capping layer is constructed to form a 2D/3D heterostructure by systematically regulating the concentration of the PEABr post-treatment solution. An optimal PEABr concentration of 1 mg/mL is identified for the formation of the desired surface 2D capping layer. The perovskite film treated with optimal PEABr exhibits the most pronounced improvements in film morphology, crystallinity enhancement, defect reduction, and suppression of nonradiative recombination. Consequently, the best-performing IPSCs achieve a champion efficiency of 24.58%, together with an open-circuit voltage of 1.15 V, a short-circuit current density of 25.68 mA/cm^2^, and a fill factor of 82.80%. This work demonstrates that regulating the PEABr concentration is an effective strategy for constructing surface 2D/3D heterostructures and achieving effective interfacial passivation, offering a promising route for advancing interface engineering and enhancing the performance enhancement of IPSCs.

## 1. Introduction

Organic/inorganic metal halide perovskite solar cells have attracted considerable attention in recent years due to their low cost, facile solution processing, and outstanding optoelectronic properties [1,2,3,4,5]. In particular, inverted perovskite solar cells (IPSCs) with a hole transport layer as the bottom and an electron transport layer (ETL) as the top of the carrier transport layers offer several attractive advantages, including negligible hysteresis effect, low-temperature processing, compatibility with multi-junction cells, and so on [6,7,8,9]. The power conversion efficiency (PCE) of IPSCs shows rapid enhancement within a short time, increasing from 3.8% to over 27% [10,11]. However, despite the rapid improvement in efficiency, the performance advancement of IPSCs based on typical three-dimensional (3D) perovskites is still significantly limited by the influence of abundant defects inevitably produced during the spin-coating and annealing process and caused by exposure to moisture and oxygen. In particular, the rich defects at the top interface between the layer of perovskite and the ETL would form detrimental nonradiative recombination centers as well as ion migration channels, severely degrading and damaging the PCE and stability of IPSCs [12,13,14]. Therefore, it is very necessary to reduce the defect intensity at the perovskites/ETL top interface by introducing external surface treatments to further improve the performance of IPSCs [15].

Employing organic spacer cations at the surface of the perovskite layer is a popular and valuable technique to effectively alleviate nonradiative recombination losses within the devices [16,17]. To date, various organic salts have been introduced to form surface passivation for the perovskite film by post-treatment, typified by organic arylammonium salts like phenethylammonium iodide (PEAI) [18] and phenethylammonium bromide (PEABr) [19]. The molecular structure of PEABr plays an important role in the formation of 2D/3D perovskite heterostructures. The bulky phenethylammonium (PEA^+^) cation cannot readily be incorporated into the 3D perovskite lattice, but its ammonium group can interact with the inorganic lead halide framework through hydrogen bonding, facilitating the formation of a layered 2D perovskite structure at the surface [20]. Compared with aliphatic ammonium salts such as butylammonium iodide (BAI), the aromatic PEA^+^ cation can introduce additional π-π interactions, which may influence the arrangement of organic spacer cations and regulate the crystal growth of 2D perovskites [21]. Furthermore, unlike PEAI, PEABr provides Br^−^ rather than I^−^, which may modify the surface halide composition and contribute to the passivation of halide-related defects [22]. For example, Zhang et al. demonstrated that PEABr post-treatment induced the formation of a surface-localized 2D perovskite phase, establishing a 2D/3D heterointerface that reduced residual PbI_2_, passivated surface and interfacial defect states, and suppressed nonradiative recombination, thereby increasing the champion PCE from 22.3% to 23.3% [23]. Moral et al. showed that PEABr post-treatment facilitated RP-phase formation and ionic redistribution at the surface of perovskites and grain boundaries, leading to enhanced defect passivation, improved device efficiency, and a 70 mV increase in V_oc_ [24]. Yu et al. reported that PEABr post-treatment chemically passivated wide-bandgap perovskite surfaces without inducing a 2D phase, simultaneously replenishing bromide vacancies and passivating undercoordinated surface sites to reduce trap-state density and interfacial nonradiative recombination, thereby increasing the champion PCE from 16.80% to 19.29% [25]. However, there are two issues still under debate. Many reports stated that low-dimensional perovskites would form at the surface of 3D perovskites to construct 2D/3D heterostructures through the reaction of organic arylammonium salts with excess lead iodide or bulky 3D perovskites [26]; another idea is that the minimization of defect-assisted recombination at the perovskite surface originates from the direct passivation of organic arylammonium salts themselves, without the presence of the 2D or quasi-2D phase observed [27]. Normally, the formation of an overly thick 2D perovskite layer would result in increased exciton binding energy and thus impede the charge transport across the 2D/3D heterointerfaces [28]. A relatively thin layer is usually adopted for efficient carrier transport, which leads to a possibility that the absence of the 2D phase is due to the passivation layer being too thin to be detected. The thickness of the surface passivation layer mostly depends on the post-treated PEABr solution concentration. Treatment with a high concentration of PEABr solution would lead to sufficient passivation and obvious phenomenon for the change in film, but undesired current and voltage loss [29]. Treatment with a low PEABr concentration commonly fails to reach the best device performance. Therefore, a systematic investigation of the PEABr concentration is required to elucidate its influence on the formation of the surface 2D capping layer and the resulting interfacial passivation and device performance in Cs/FA/MA triple-cation perovskite solar cells.

Herein, we construct a surface 2D capping layer to form a surface 2D/3D heterostructure by systematically varying the concentration of the PEABr post-treatment solution. An optimal concentration of 1 mg/mL is identified for achieving effective surface modification and interfacial passivation. The perovskite film subjected to this optimal PEABr treatment exhibits the most pronounced improvements in film morphology, crystallinity enhancement, defect reduction, and suppression of nonradiative recombination. As a result, devices based on the optimal PEABr treatment deliver the highest photovoltaic performance, yielding a champion PCE of 24.58%, along with a Voc of 1.15 V, a Jsc of 25.68 mA/cm^2^, and an FF of 82.80%. Collectively, this work not only provides a systematic understanding of concentration-dependent passivation effects but also offers valuable insights for developing more effective interfacial passivation strategies in IPSCs.

## 2. Materials and Methods

Materials: Formamidinium iodide (FAI, 99.5%), phenethylammonium bromide (PEABr, 99.5%), and bathocuproine (BCP, >98%) were supplied by Greatcell Solar Materials Pty Ltd. (Queanbeyan, Australia); methylammonium iodide (MAI, 99.9%) was acquired from Advanced Election Technology Co., Ltd. (Shenyang, China); cesium iodide (CsI, 99.99%), lead iodide (PbI_2_, 99.99%), methylammonium chloride (MACl, 99.99%), and phenyl-C61-butyric acid methyl ester (PC_61_BM, 99%) were purchased from Xi’an Polymer Light Technology Corp. (Xi’an, China); (4-(3,6-dimethyl-9H-carbazole-9-yl) butyl)phosphonic acid (Me-4PACz, 99%) was purchased from TCI (Tokyo, Japan); and chlorobenzene (CB, 99.99%, anhydrous), N,N-dimethylformamide (DMF, >99.9%, anhydrous), and isopropyl alcohol (IPA, 99.5%, anhydrous), as well as dimethyl sulfoxide (DMSO, >99.8%, anhydrous), were sourced from Sigma-Aldrich (St. Louis, MO, USA). All the materials were obtained commercially and used as received without further purification.

Device Fabrication: Firstly, ITO-coated glass substrates were sequentially ultrasonicated in deionized water, acetone, and isopropyl alcohol for 15 min each, respectively, followed by drying with nitrogen. The cleaned ITO substrates were treated with ultraviolet-ozone for 20 min before film deposition. Then, all substrates were transferred into a nitrogen-filled glove box, and the Me-4PACz HTLs solution (0.5 mg/mL in the isopropyl alcohol/DMF, *v*/*v* = 50:1) was spin-coated onto ITO substrates at 4000 rpm for 30 s and then annealed at 100 °C for 10 min. The 1.65 M Cs_0.05_FA_0.85_MA_0.1_PbI_3_ precursor was prepared by adding 26.2 mg MAI, 21.4 mg CsI, 241.2 mg FAI, 824 mg PbI_2_, and 13.5 mg MACl into 1 mL DMF/DMSO mixed solution (volume ratio of 4:1), which was prepared and then was stirred for 2 h. For the deposition of the perovskite layer, 50 μL of perovskite precursor was spin-coated on the Me-4PACz substrates at 1000 rpm for 10 s and then at 5000 rpm for 26 s. A total of 200 μL of chlorobenzene anti-solvent was quickly dripped onto the film at 13 s before the end of the whole program and then it was annealed at 100 °C for 30 min. For the control device, no post-treatment was applied. For the samples with PEABr post-treatment, different concentrations (0.5 mg, 1 mg, or 1.5 mg in a mixed solution of 995 μL IPA and 5 μL DMSO) of PEABr solutions were dynamically spin-coated at 4000 rpm for 35 s and annealed at 100 °C for 5 min. Then, 20 mg/mL PCBM in chlorobenzene was spin-coated at 4000 rpm for 30 s. Lastly, 8 nm of BCP and a 100 nm silver electrode were evaporated under high vacuum in sequence.

Characterizations and Device Measurement: Scanning Electron Microscopy (SEM) images were obtained using a field-emission scanning electron microscope (Thermo Scientific Apreo C, Waltham, MA, USA). Ultraviolet–visible spectroscopy (UV–vis) spectra were conducted using a Shimadzu UV-3600 UV–vis–NIR spectrophotometer (Kyoto, Japan). Photoluminescence (PL) and time-resolved photoluminescence (TRPL) were measured using a PicoQuant FluoTime 300 instrument (Berlin, Germany) with an excitation of 405 nm. X-ray diffraction (XRD) was tested via a Bruker D8 ADVANCE (Karlsruhe, Germany). The current density–voltage (J-V) characteristics were tested using a Keithley 2400 source meter (Solon, OH, USA) under a simulated AM 1.5G illumination (100 mW∙cm^−2^) by a solar simulator (Wacom Denso Co., Ltd., Kariya, Japan). The external quantum efficiency (EQE) was measured using a quantum efficiency instrument (QE-R, Enli Technology Co., Ltd., Kaohsiung, Taiwan). The Atomic Force Microscopy (AFM) measurements were conducted via a Bruker Dimension Icon (Santa Barbara, CA, USA).

## 3. Results and Discussion

**Figure 1:**
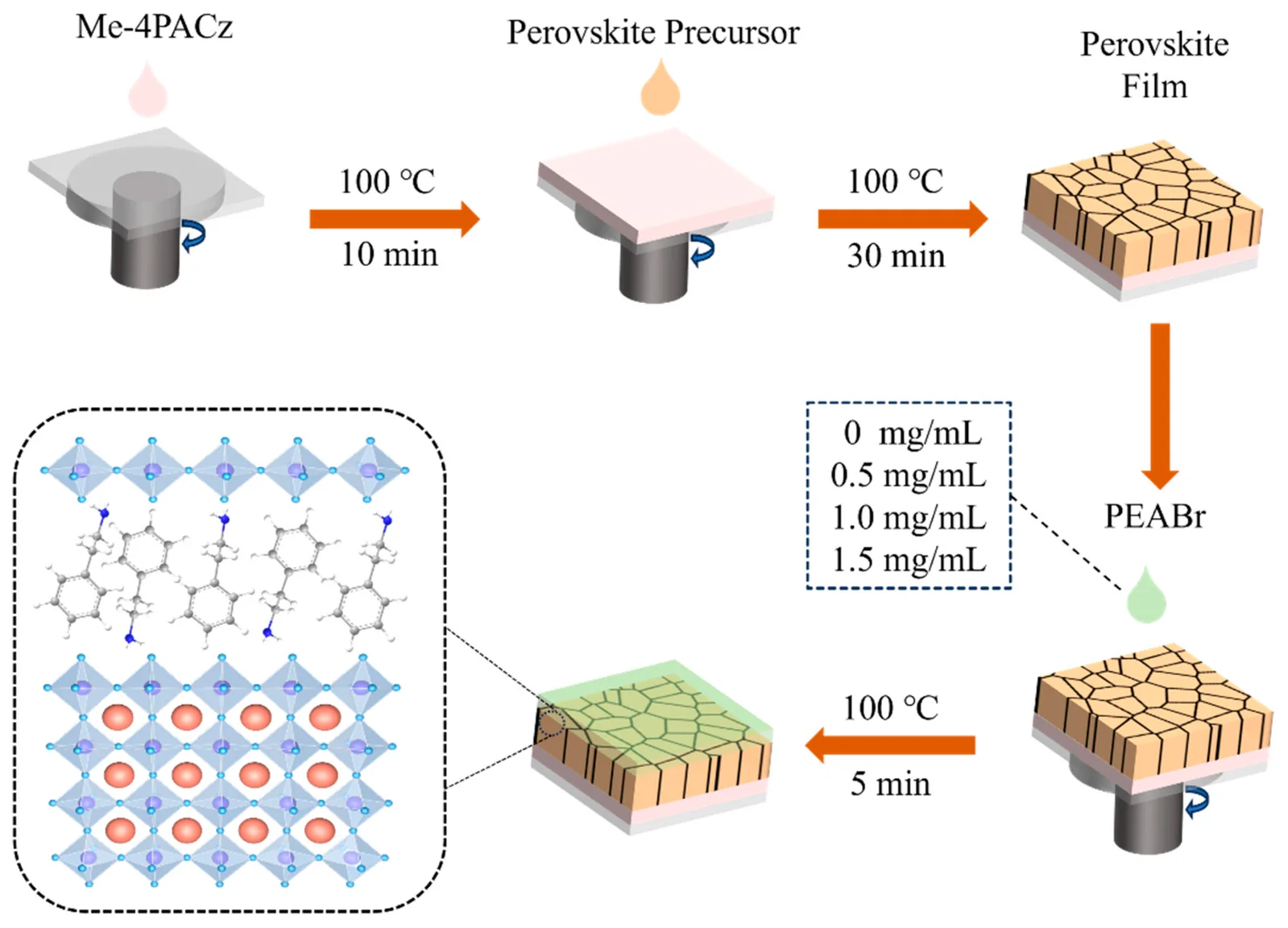
Schematic illustration of the film-deposition processes.

The device fabrication process is presented in Figure 1. Following the deposition of the perovskite film onto the Me-4PACz/ITO/glass substrate, different concentrations of PEABr solutions were applied to provide tailored post-treatment of the perovskite film. The treated PEABr significantly changed the perovskite morphology and possibly led to the formation of a 2D/3D heterostructure on the top surface of the perovskite film, which will be discussed in detail later. The device was constructed with a structure of ITO/Me-4PACz/perovskites/post-treated PEABr/PC_61_BM/BCP/Ag. To form the desired surface passivation layer and achieve significantly enhanced efficiency of IPSCs, we systematically adjusted the concentration of the PEABr post-treatment solution. Devices treated with PEABr concentrations of 0, 0.5, 1, and 1.5 mg/mL were fabricated, respectively. The complete experimental processes are described in Section 2.

**Figure 2:**
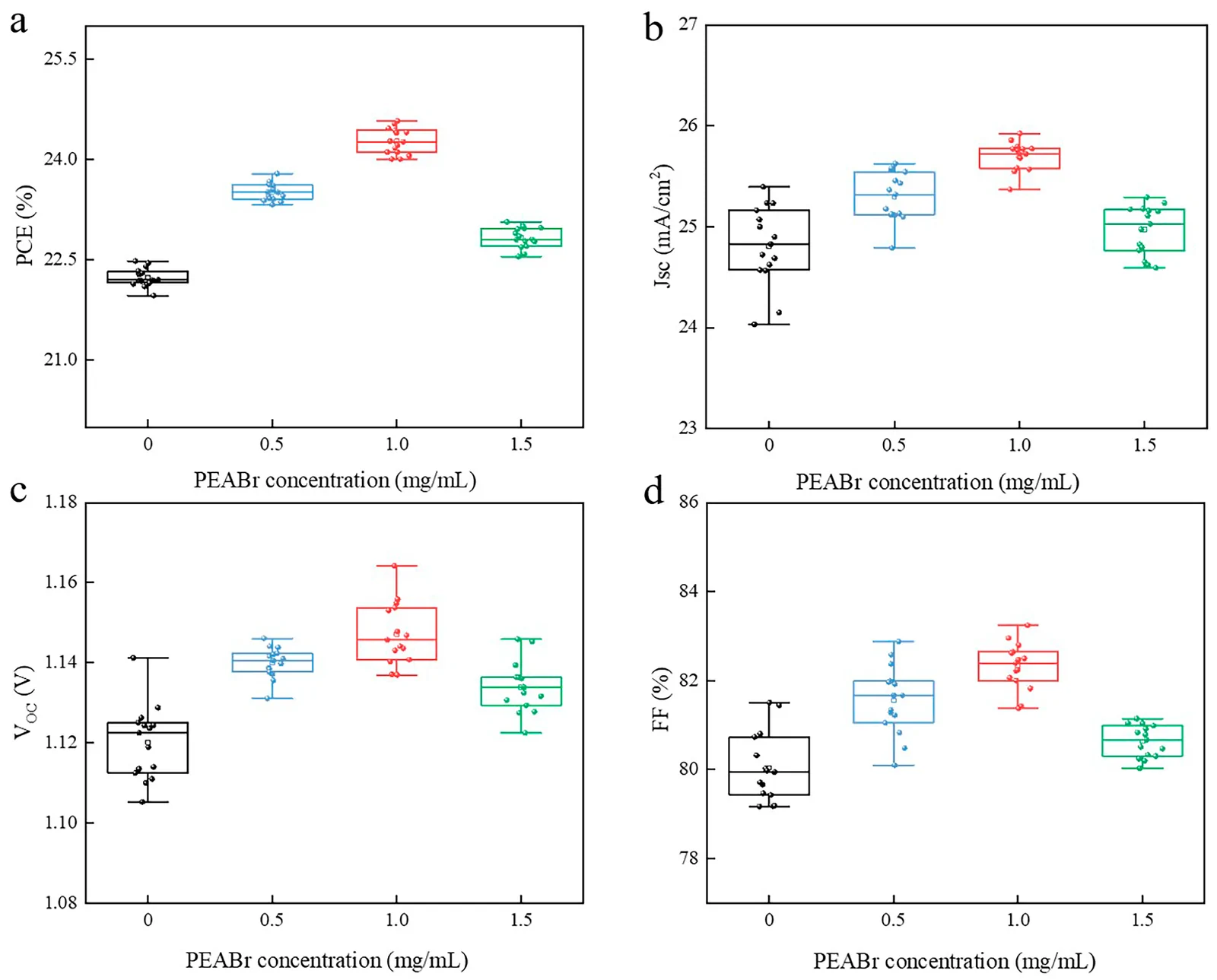
Statistical distributions of (**a**) PCE, (**b**) Jsc, (**c**) Voc, and (**d**) FF of IPSCs based on different concentrations of PEABr post-treatment solution.

To investigate the influence of varied concentrations of PEABr on the photovoltaic performance of IPSCs and to determine the optimal PEABr concentration, we firstly measured the photovoltaic parameters, including open-circuit voltage (Voc), short-circuit current density (Jsc), fill factor (FF), and PCE, for 15 devices fabricated under identical conditions at each PEABr concentration. The statistical distributions of the device performance are shown in Figure 2. Clearly, compared with control devices (i.e., devices with 0 mg/mL PEABr), IPSCs treated with different concentrations of PEABr show an obvious increase in device performance. All of the device performances of Voc, Jsc, FF and PCE gradually increase when the concentration of the PEABr post-treatment solution increases to 0.5 mg/mL and 1 mg/mL. When the concentration of PEABr further increases to 1.5 mg/mL, the photovoltaic parameters of the device begin to drop, leading to decreased PCE values. Therefore, the optimal concentration for the PEABr post-treatment solution is determined and evaluated to be 1 mg/mL. Accordingly, the IPSCs with the optimal 1 mg/mL PEABr treatment show the highest average PCE of 24.27%, along with a high average Voc of 1.147 V, an average FF of 82.32%, and an average Jsc of 25.70 mA/cm^2^. The specific values of device performance parameters are listed in Table 1.

**Figure 3:**
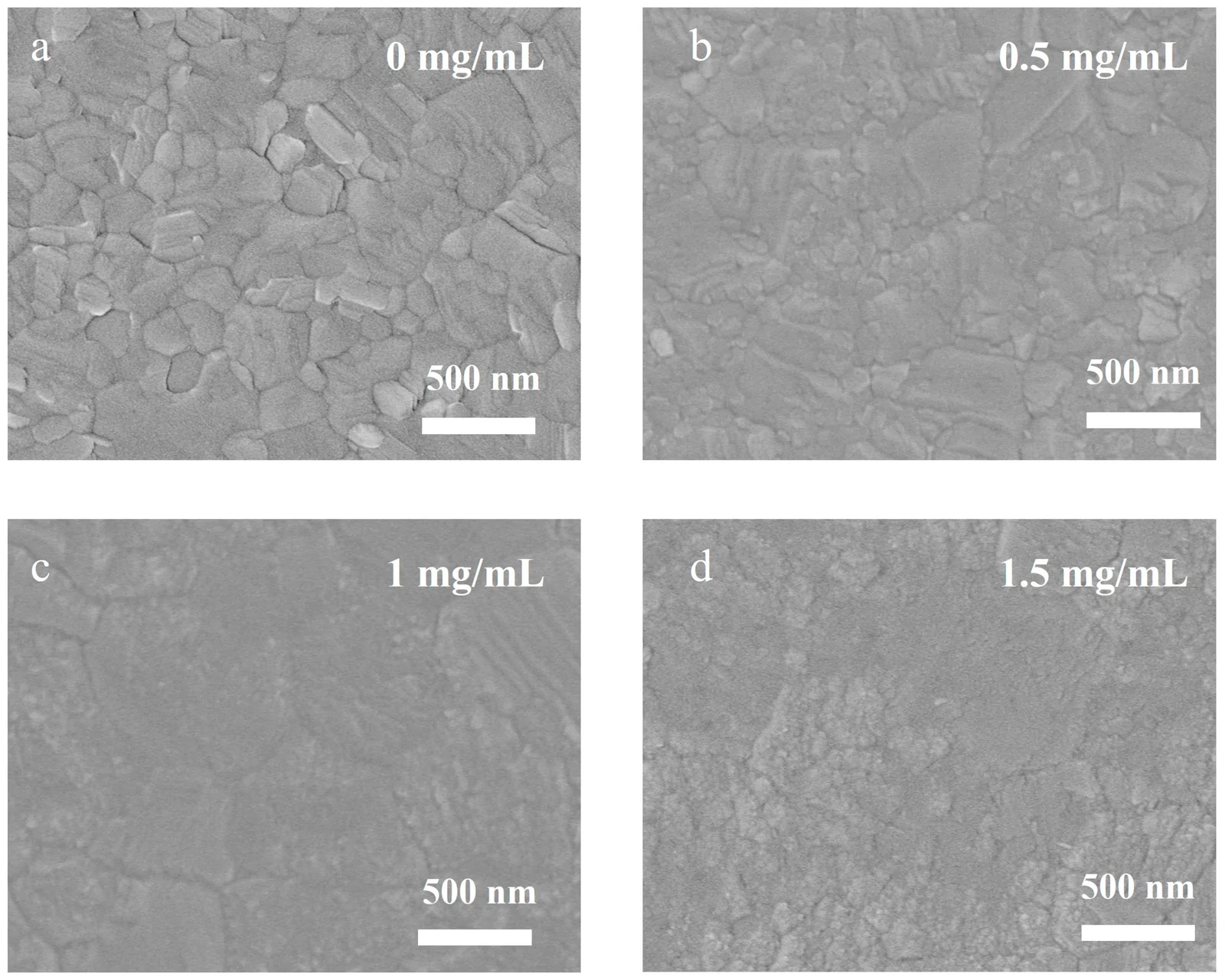
SEM images of perovskite films treated with (**a**) 0 mg/mL, (**b**) 0.5 mg/mL, (**c**) 1 mg/mL, and (**d**) 1.5 mg/mL PEABr post-treatment solution.

It is suggested that the highest efficiency achieved in the device with 1 mg/mL PEABr post-treatment solution is due to the optimal concentration, which leads to the desired surface morphology of the perovskite film and forms the most suitable capping layer on the film surface, contributing to the significantly decreased defects and increased device performance. Therefore, we then explored the influences of different concentrations of PEABr post-treatment solution on the film morphology. The perovskite film surfaces present large changes after being treated with PEABr. In the control perovskite film (i.e., film with 0 mg/mL PEABr), the surface grains show two distinguished regions of white and dark, which indicate the presence of two phases on the perovskite surface (as shown in Figure 3a). The dark grain region corresponds to the perovskite phase, while the bright region corresponds to the PbI_2_ phase [30]. The control film surface exhibits an uneven grain distribution, with pronounced undulations between adjacent grains. After being treated with PEABr, it is obvious that the surface microstructure of the films shows obvious changes. When the concentration of PEABr increases from 0 mg/mL to 0.5 mg/mL and then to 1.0 mg/mL, the grain boundaries on the perovskite surface gradually decrease and become less distinct, as if the grains are progressively fusing together (Figure 3b,c). As the PEABr concentration is further increased to 1.5 mg/mL, the grain boundaries become nearly indistinguishable, and a thicker capping layer is observed on the perovskite film surface (Figure 3d). The above phenomenon is most probably attributed to the fact that the exposed 3D perovskite grains are covered by the formed 2D perovskite materials, which leads to 2D/3D heterostructures at the film surface, serving as a blocking layer for moisture and oxygen [31].

To further investigate the surface morphology of the perovskite films, atomic force microscopy (AFM) measurements were performed, as shown in Figure S1 in the Supporting Information. The root-mean-square roughness (Rq) decreases from 24.6 nm for the control film to 18.4 nm and 14.6 nm after treatment with 0.5 and 1.0 mg/mL PEABr, respectively. When the PEABr concentration is further increased to 1.5 mg/mL, the Rq slightly increases to 15.5 nm. These results demonstrate that PEABr treatment effectively reduces the surface roughness, with the lowest Rq obtained at the optimal concentration of 1.0 mg/mL. The improved surface smoothness is consistent with the morphological changes observed in the SEM images, further confirming the beneficial effect of PEABr treatment on the perovskite film morphology. Moreover, the target perovskite film treated with the optimal PEABr concentration of 1 mg/mL shows the smoothest, most compact, and densest surface, which would effectively contribute to reducing defect density and enhancing interface contact.

**Figure 4:**
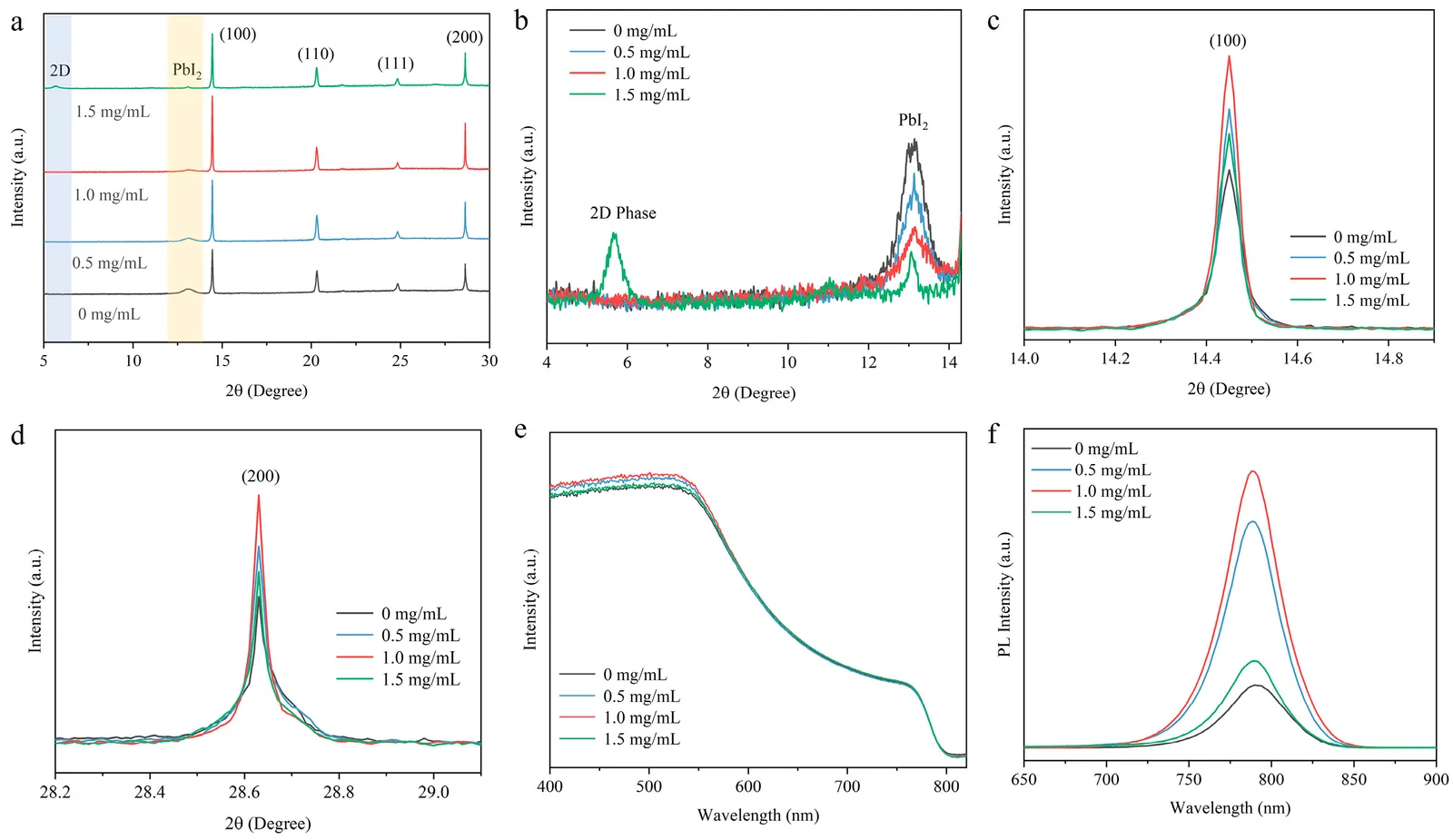
(**a**,**b**) XRD patterns, (**c**) (100) diffraction peaks, (**d**) (200) diffraction peaks, (**e**) absorption curves and (**f**) PL curves of perovskite films treated with different PEABr concentrations.

Furthermore, XRD measurements were conducted to determine the crystal structure of the perovskite films with different concentrations of PEABr post-treatment solution. As presented in Figure 4a, the peak positions of (100), (110), (111), and (200) belonging to perovskites for different samples are identical, indicating that the crystal phases of the perovskite films remain unchanged after PEABr treatment. However, as clearly shown in Figure 4b, the peak intensity of PbI_2_ shows a gradual decrease with the increased concentration of PEABr post-treatment solution, suggesting that PEABr can react with the excess PbI_2_ at the surface of the perovskite film to form a thin 2D layer. Correspondingly, a 2D phase is clearly observed in the perovskite sample with 1.5 mg/mL PEABr, evidencing the formation of a 2D perovskite capping layer on the top of 3D perovskites and thus 2D/3D heterostructures. Although no distinct 2D diffraction peak is detected in the XRD patterns of perovskite films treated with 0.5 and 1.0 mg/mL PEABr, the presence of a small amount of low-dimensional perovskite species at the film surface cannot be excluded, as their diffraction signals may fall below the detection limit of conventional XRD [32]. Additionally, it is clearly observed that compared with the control perovskite film, the intensities of the (100) and (200) crystal planes for the perovskite film treated by optimal 1 mg/mL PEABr demonstrate substantial increases (Figure 4c,d), which indicates the effectively enhanced crystallinity of perovskites.

We then employed light absorption spectroscopy and photoluminescence to investigate the influence of the generated 2D/3D heterostructure capping layer on the optoelectronic properties of perovskite films. As demonstrated in Figure 4e, the absorption edges of the control perovskite film and the films with different concentrations of PEABr are nearly identical, indicating that the post-treatment does not affect the bandgaps of perovskites. Surprisingly, the absorption intensity around the wavelength of 400–550 nm of perovskite treated with optimal PEABr is obviously higher than that of the control film, which suggests the promoted crystallinity of the film and is in agreement with the XRD results. The tauc plots for the bandgaps calculated from the absorption spectra are shown in Figure S2 in the Supporting Information. In addition, compared to the control perovskite film, perovskite films treated with different concentrations of PEABr show enhanced PL intensities, and that treated with the optimal concentration of 1 mg/mL PEABr presents the highest intensity value, as clearly presented in Figure 4f. This result confirms the effectively decreased surface defects for the perovskite film and thereby the largely inhibited nonradiative recombination by the formed 2D/3D capping layer. The PL peaks of perovskite films treated with different concentrations (i.e., 0.5, 1, and 1.5 mg/mL) of PEABr show slightly blueshift behavior in comparison with the control film, which can be attributed to the suppression of low-energy defect-related emission by surface passivation [33,34]. Moreover, we also measured the PL of samples of perovskite films with the ETL (e.g., glass/perovskites/PCBM), as shown in Figure S3 in the Supporting Information. The PL intensity decreases significantly upon PEABr treatment when the perovskite film is in contact with PCBM, and, importantly, PL quenching is most pronounced in the sample with the optimal PEABr treatment, which indicates improved interfacial contact and thus enhanced charge transfer from the perovskite to the ETL.

**Figure 5:**
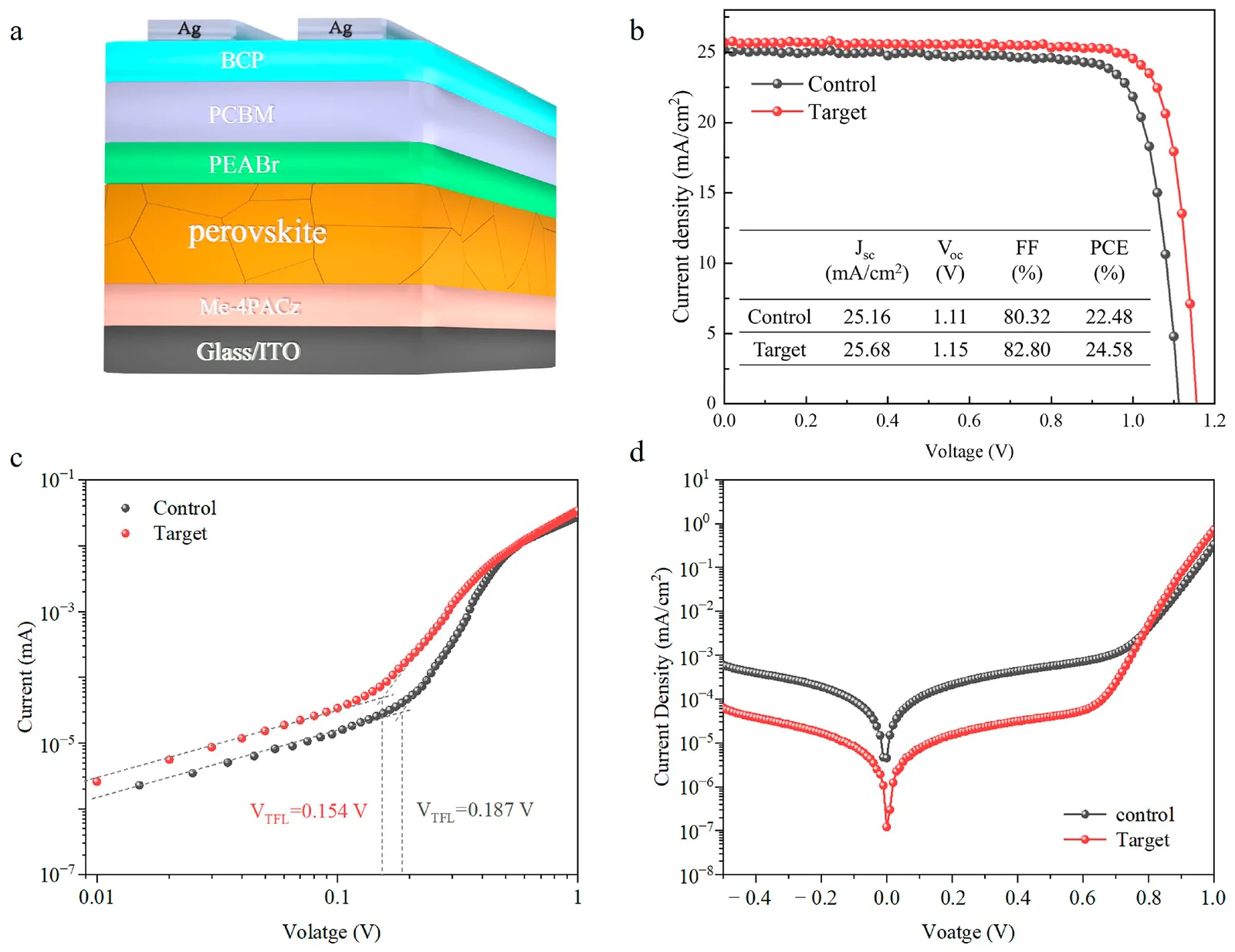
(**a**) Illustration of target device structure, (**b**) J-V curves of the champion devices, (**c**) SCLC measurement for control and target samples, (**d**) dark currents of the control and target IPSCs.

Based on the above results, we found that the 3D perovskite film with the desired capping layer resulting from the optimal 1 mg/mL PEABr post-treatment solution presents the most improved surface morphology, enhanced crystallinity, and suppressed trap density, as well as nonradiative recombination. Therefore, the performance of IPSCs without (control device) and with optimal 1 mg/mL PEABr (target device) was further studied by examining J-V curves and dark currents, and we further verified the statistical distributions of the photovoltaic parameters. Figure 5a illustrates the device architecture of the fabricated IPSCs with the configuration of ITO/Me-4PACz/Cs_0.05_FA_0.85_MA_0.1_PbI_3_/PEABr/PC_61_BM/BCP/Ag. Compared with the control device with a PCE of 22.48%, a Voc of 1.11 V, a Jsc of 25.16 mA/cm^2^, and an FF of 80.32%, the target IPSCs exhibit a much higher performance, showing a champion PCE of 24.58%, accompanied by a Voc of 1.15 V, a Jsc of 25.68 mA/cm^2^, and an FF of 82.80% (as depicted in Figure 5b). Furthermore, the forward and reverse J-V curves of the champion device exhibit PCE values of 24.48% and 24.58%, respectively (Figure S4, Supporting Information), indicating minimal J-V hysteresis. The EQE spectra of the control and target devices are shown in Figure S5 in the Supporting Information, with integrated current densities of 24.25 and 24.69 mA/cm^2^, respectively, compared with the corresponding Jsc values of 25.16 and 25.68 mA/cm^2^, obtained from the J-V measurements. The increased integrated current density of the target device further confirms the improvement in photocurrent generation following PEABr treatment.

Moreover, space-charge-limited current (SCLC) measurement is performed using electron-only devices with the configuration of ITO/SnO_2_/perovskite (with or without PEABr post-treatment)/PCBM/Ag to evaluate the trap-state density by extracting the trap-filled limit voltage (V_TFL_), as shown in Figure 5c. The trap-state density is calculated using the following equation [35]:

$$N_{t}=\frac{2\varepsilon_{0}\varepsilon_{r}V_{\mathrm{TFL}}}{qL^{2}}$$

where ε_0_ is the vacuum permittivity, ε_r_ is the relative dielectric constant, q is the elementary charge, and L is the perovskite film thickness. In comparison with the control device which shows a V_TFL_ of 0.187 V, the target device with the perovskite film treated with the optimal PEABr concentration exhibits a markedly lower V_TFL_ of 0.154 V, with the corresponding trap density decreasing from 6.69 × 10^14^ to 5.51 × 10^14^ cm^−3^. This finding indicates that the 2D capping layer formed by the optimal PEABr treatment effectively suppresses trap formation within the perovskite active layer, which finally leads to the much higher device performance of IPSCs. The decreased trap density for the target perovskite treated with the optimal PEABr concentration is also evidenced by its largely prolonged charge carrier lifetime (Figure S6, Supporting Information), further confirming the suppressed defects and nonradiative recombination [36]. Finally, we test the dark current curves of control and target IPSCs. As presented in Figure 5d, the dark current density of the target device is largely decreased, being over 1 order of magnitude lower than that of the control device, which further confirms the significantly reduced leakage and suppressed nonradiative recombination resulting from effective passivation and reduced defect density due to the desired 2D capping layer formed by the optimal PEABr post-treatment. In addition, the storage stability of unencapsulated IPSCs was evaluated in a nitrogen atmosphere (RH < 5%) at room temperature. As shown in Figure S7 in the Supporting Information, the target device retains approximately 97% of its initial PCE after 216 h, decreasing from 24.58% to 23.86%, whereas the control device retains only 89%, decreasing from 22.48% to 20.07%, demonstrating improved storage stability after optimal PEABr treatment.

## 4. Conclusions

In summary, we systematically adjusted the concentration of the PEABr post-treatment solution to achieve a desired 2D capping layer and thus construct a 2D/3D heterostructure at the surface of the perovskites to significantly increase the performance of IPSCs. By regulating the PEABr concentration, the optimal ratio was determined to be 1 mg/mL. The perovskite film treated with the optimal PEABr concentration showed the greatest improvements in film morphology and crystallinity, as well as decreased trap density and suppressed nonradiative recombination. Consequently, the highest photovoltaic parameters were achieved by the devices with the optimal PEABr treatment. The target IPSCs achieved a champion PCE of 24.58%, along with a Voc of 1.15 V, a Jsc of 25.68 mA/cm^2^, and an FF of 82.80%. This work provides clear evidence of concentration-dependent passivation effects in photovoltaics and offers support for advancing potential methods for effective interface passivation of IPSCs.

## Figures and Tables

**Table 1:** Average performance of IPSCS with different concentrations of PEABr post-treatment solution.

Concentration (mg/mL)	Jsc (mA/cm^2^)	Voc (V)	FF (%)	PCE (%)
0	24.81	1.120	80.04	22.24
0.5	25.29	1.140	81.56	23.53
1.0	25.70	1.147	82.32	24.27
1.5	24.97	1.134	80.63	22.83

## Data Availability

Data is contained within the article or Supplementary Material.

## Supplementary Materials

The following supporting information can be downloaded at https://www.mdpi.com/article/10.3390/nano16211445/s1. Figure S1: AFM images of perovskite films treated with different PEABr concentrations: (a) 0 mg/mL, (b) 0.5 mg/mL, (c) 1.0 mg/mL, and (d) 1.5 mg/mL; Figure S2: Tauc plots for the perovskite films with different concentrations of post-treated PEABr; Figure S3: PL of samples with a structure of glass/perovskites/PCBM upon different concentration of PEABr post-treatment; Figure S4: Forward and reverse J-V curves of the champion device; Figure S5: EQE spectra of the control and champion devices; Figure S6: TRPL curves with fitted parameters of control and target perovskite films. The TRPL curves were fitted according to the biexponential decay function of I(t) = I_0_ + A_1_exp(−t/τ_1_) + A_2_exp(−t/τ_2_); Figure S7: PCE stability of unencapsulated IPSCs stored in a nitrogen atmosphere (RH < 5%) at room temperature for 216 h.
