# Supplementary material for: Optimization of Phenylethylammonium Bromide Post-Treatment for 2D/3D Heterostructure Formation in High-Performance Perovskite Solar Cells
Source: Nanomaterials (Basel). 2026 Nov 8;16(21):1445. doi: 10.3390/nano16211445 (PMC13764809; doi:10.3390/nano16211445)
Supplement: Supplementary file 1 [file nanomaterials-16-01445-s001.zip › nanomaterials-4675876-supplementary.pdf]

Optimization of Phenylethylammonium Bromide Post-Treatment for 2D/3D Heterostructure Formation in High-Performance Perovskite Solar Cells

Hui Liu*,†, Guoquan Wu†, Junqing Mo, Ruiheng Zhang

School of Physics and Opto-Electronic Engineering, Guangdong Provincial Key Laboratory of Sensing Physics and System Integration Applications, Guangdong University of Technology, Guangzhou 510006, China

* Corresponding Author, E-mail: liuhui@gdut.edu.cn

†H. Liu and G. Wu contribute equally to this work.

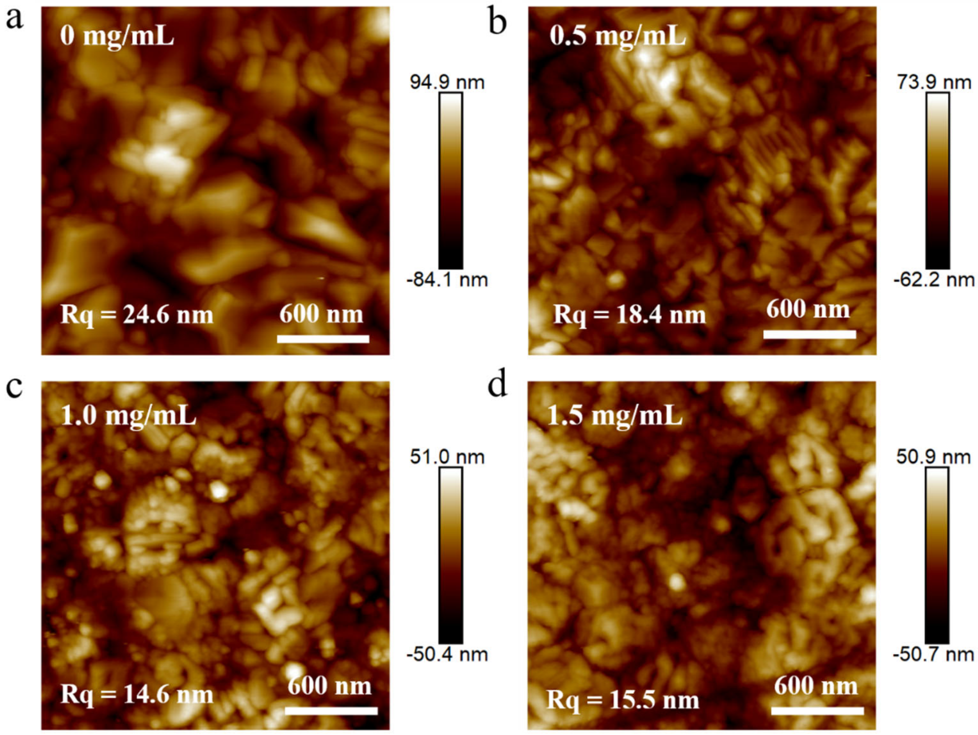

Figure S1. AFM images of perovskite films treated with different PEABr concentrations: (a) 0 mg/mL, (b) 0.5 mg/mL, (c) 1.0 mg/mL, and (d) 1.5 mg/mL.

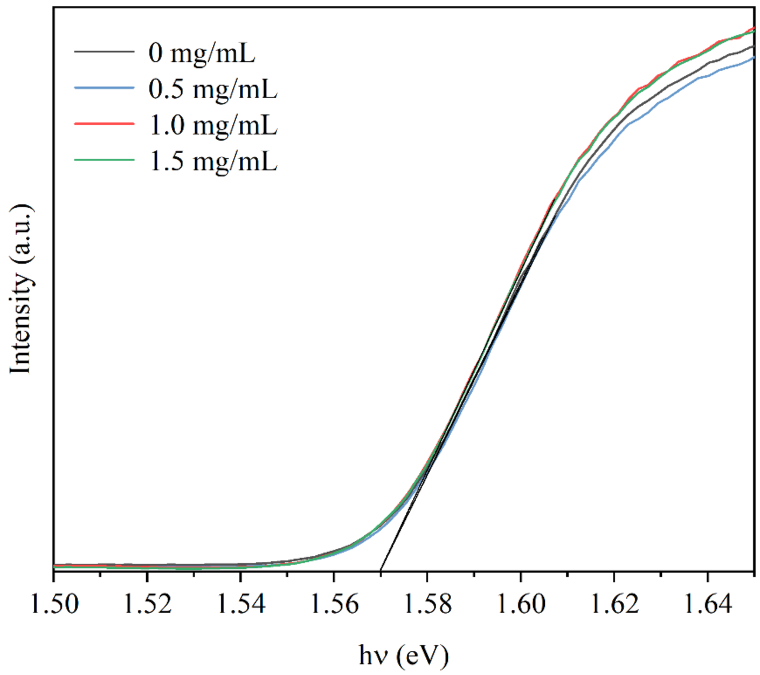

Figure S2. Tauc plots for the perovskite films with different concentrations of post-treated PEABr.

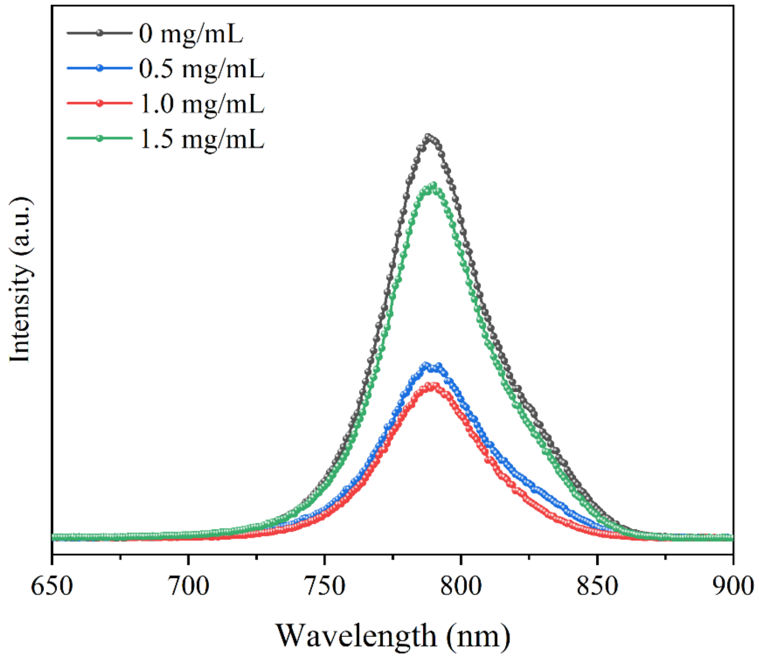

Figure S3. PL of samples with a structure of glass/perovskites /PCBM upon different concentration of PEABr post-treatment.

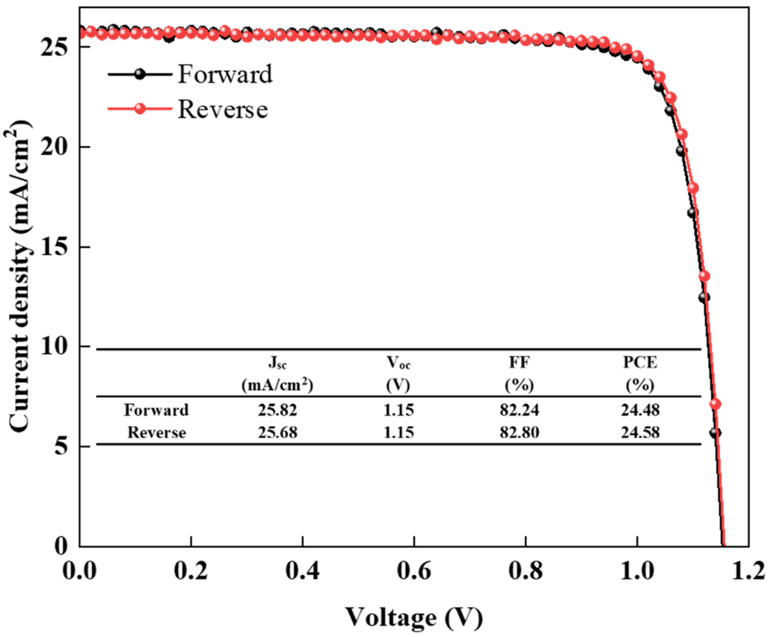

Figure S4. Forward and reverse J-V curves of the champion device.

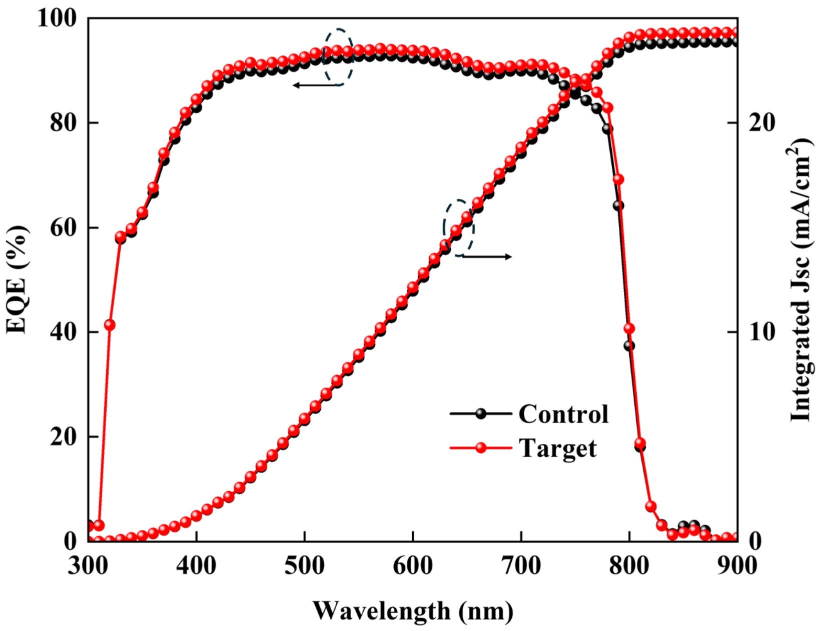

Figure S5. EQE spectra of the control and champion devices.

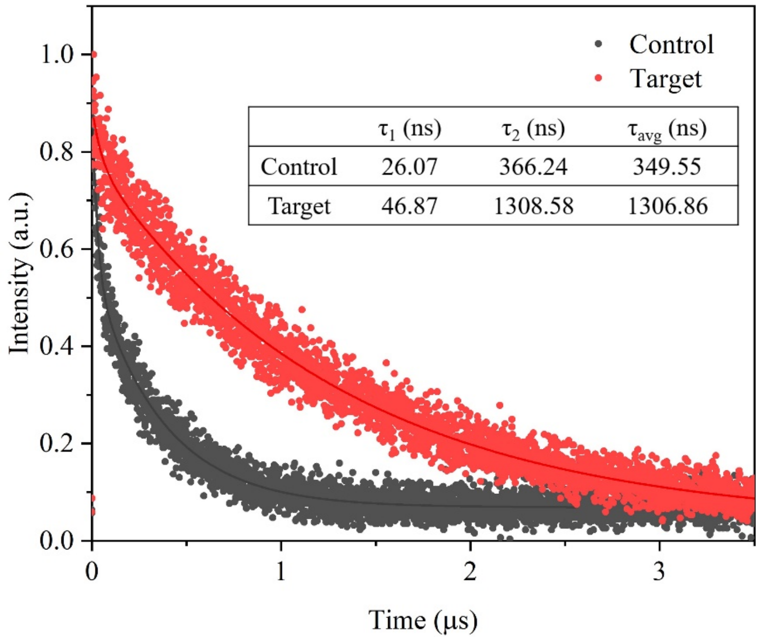

Figure S6. TRPL curves with fitted parameters of control and target perovskite films. The TRPL curves were fitted according to the biexponential decay function of $I(t) = I_0 + A_1\exp(-t/\tau_1) + A_2\exp(-t/\tau_2)$.

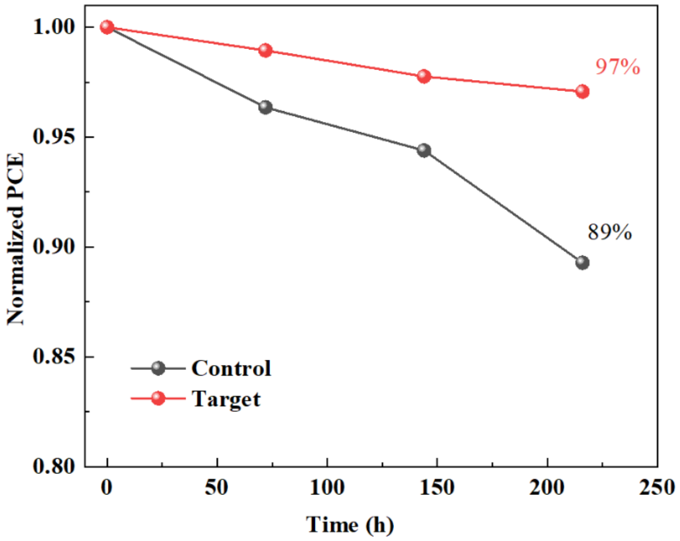

Figure S7. PCE stability of unencapsulated IPSCs stored in a nitrogen atmosphere (RH < 5%) at room temperature for 216 h.
